# Desmoglein-3 autoantibodies in tissues of oral lichen planus patients and its correlation with disease severity: case-control study

**DOI:** 10.1186/s12903-025-06303-9

**Published:** 2025-06-06

**Authors:** Doaa Abdelwadood, Yasmine Ahmed Fouad, Nashwa El-Khazragy, Ahmed Elsayed Hamed Amr

**Affiliations:** 1 Faculty of Dentistry, Department of Oral Medicine, Periodontology, and Oral Diagnosis, Ain Shams University, Cairo, 11566 Egypt; 2 Associate Professor of Oral Medicine, Periodontology, and Oral Diagnosis, Faculty of Dentistry, Ain Shams University, Cairo, Egypt; 3 Faculty of Medicine, Department of Clinical Pathology-Hematology and Ain Shams Medical Research Institute (MASRI), Ain Shams University, Cairo, 11566 Egypt; 4 Assistant Lecturer at Oral Medicine, Periodontology, and Oral Diagnosis Department, Faculty of Dentistry, Ahram Canadian University, Giza, Egypt

**Keywords:** Oral lichen planus, Desmogleins, Autoantibodies, Atrophic\bullous erosive

## Abstract

**Background:**

Due to the not fully understood exact pathogenesis of oral lichen planus, the patients receive symptomatic management, rather than a curative treatment. Consequently, revealing the pathogenesis of OLP is a primary concern in oral medicine research. Elevated levels of circulating antibodies against Desmoglein 3 have been discovered in the serum of OLP patients. The aim of the present study was to evaluate the level of Desmoglein 3 autoantibodies in tissue biopsies of atrophic/bullous erosive OLP and to correlate it with the disease severity, in attempt to reveal if it has a role in the pathogenesis of the disease.

**Methods:**

This is a case-control study, tissue biopsies were obtained from clinically and histopathologically diagnosed atrophic/bullous-erosive oral lichen planus (OLP) lesions (*n* = 10). The oral lichen planus biopsies were compared with healthy uninflamed gingival tissues excised during periodontal surgeries (*n* = 10). The tissue biopsies were tested for quantitative levels of desmoglein 3 autoantibodies using ELISA test. The clinical severity of oral lichen planus lesions was evaluated by Elsabagh scoring system. The levels of desmoglein 3 autoantibodies were correlated with the disease severity.

**Results:**

The concentration of desmoglein 3 autoantibodies level in tissues of patients with atrophic/erosive OLP [3395.4 (± 526.9) Pg/g] was significantly higher (*p* < 0.001) than in tissues of healthy controls [2329.7 (± 307.6) Pg/g]. The student’s t-test was used to compare between the two groups. Moreover, the concentration of desmoglein 3 autoantibodies showed a statistically significant positive correlation (ρ = 0.801) with OLP clinical severity scores (*p* = 0.005).

**Conclusions:**

Desmoglein 3 autoantibodies were detected in higher concentrations in oral lichen planus tissues compared to healthy controls. Increased concentration of desmoglein 3 autoantibodies is correlated with an increase in oral lichen planus clinical severity scores and vice versa. So, further investigation is needed to discover the exact role of Dsg3 autoantibodies in the pathogenesis of OLP.

**Trial registration:**

The study was registered on the Clinical Trial Registration Site (registration code: **NCT**06652477, last updated on 22-10-2024).

## Background

Oral lichen planus (OLP) is a chronic immune-mediated disease affecting oral mucosa of 0.5–2% of the general population. The disease has female predilection and usually affects middle-aged individuals [1, 2]. Oral lichen planus has diverse clinical presentations with variable subtypes. The atrophic-erosive subtype is usually associated with pain and burning sensation. Besides, the lesions have a chronic course and tend to progress with time [3]. Therefore, it disrupts the patient’s quality of life to an extent that may lead to serious consequences [4].

Unfortunately, owing to the not fully discovered exact etiology and pathogenesis of OLP, the patients receive symptomatic management; rather than a curative treatment [5]. Therefore, revealing the pathogenesis of OLP represents a primary concern for oral medicine research.

Several mechanisms are postulated in the immune pathogenesis of OLP. The cell-mediated immune response is one of the theories that play a major role in the pathogenesis of OLP. The human normal epidermis has a low percentage of lymphocytes, generally in the basement membrane (BM). The lymphocytic infiltrate in lesions of OLP consists mainly of T cells, (CD 4 + and CD8 + lymphocytes), that migrate to the epithelium [6]. When the CD8 + T cells are activated, it kills the basal keratinocytes through TNF-α [7]. Also, one of the mechanisms postulated in the immune pathogenesis of OLP is the role of humoral immunity [6]. This role was based on detecting B lymphocytes and plasma cells in the oral mucosa and skin affected by LP [8, 9]. B lymphocytes, a white blood cell responsible for producing antibodies, have been found to play a role in the development of this condition. An Immunohistochemistry analysis has shown the presence of B lymphocytes and plasma cells in the affected oral mucosa and skin of OLP patients. This suggests that the production of antibodies by activated B lymphocytes contributes to the inflammatory response seen in OLP [8, 9]. Moreover, a recent study on OLP patients’ serum showed significant changes in antibody concentrations, with increased IgA levels and decreased IgM, IgE, and complement C3 and C4 components [10]. Elevated levels of specific antibodies and alterations in serum antibody concentrations indicate the involvement of B lymphocytes and humoral immunity in the pathogenesis of OLP.

Furthermore, high levels of circulating antibodies were discovered in the serum of OLP patients [6]; including antibodies against Desmoglein 1 (Dsg1) and Desmoglein 3 (Dsg3) [11, 12] Desmoglein proteins are crucial for maintaining cellular cohesion in the epidermis and mucosal surfaces. The Dsg3 antigen is located in the basal membrane zone, preventing cells from detachment [12].

A recent review was concerned with the detection of autoantibodies directed against Dsg1 and 3 in patients with OLP. The review concluded that although circulating Dsg1 and 3 autoantibodies were reported to be detected in patients with OLP, the scientific literature on this topic is limited to retrospective studies and case reports [12].

However, all the studies targeted detecting Dsg3 autoantibodies in the serum of OLP patients. It is difficult to infer whether these circulating autoantibodies increased as an etiologic agent in OLP or due to inflammatory damage to basal keratinocytes. The damage causes the release of Dsg3 proteins, which serve as autoantigens, triggering the release of autoantibodies into the bloodstream [2, 13].

Thus, the current study aims to detect the level of Dsg3 autoantibodies in tissues affected by atrophic/bullous erosive OLP and correlate it with the disease severity.

## Methods

### Study design

The current study was designed to be a case-control study that included ten patients suffering from atrophic/bullous-erosive (A/BE) forms of oral lichen planus and ten healthy controls. Participants were recruited from the outpatient clinic of the Oral Medicine, Periodontology, and Oral Diagnosis Department at the Faculty of Dentistry, Ain Shams University in Egypt. All participants were fully informed about the purpose of the study and provided their consent by signing an informed consent form. The study was conducted in accordance with the Declaration of Helsinki and its amendments.

### Study sample

The study included a convenience sample of twenty participants, allocated as follows:

- Group A (*n* = 10): Patients with clinically and histologically diagnosed symptomatic oral lichen planus (atrophic/bullous-erosive forms) [14–16].
- Group B (*n* = 10): Healthy control participants had no oral or skin lesions of LP or any other oral lesion affecting the oral mucosa.

Group A patients were enrolled in the study if they were classified as ASA class II [17] and suffered from atrophic/erosive forms of OLP with confirmed diagnosis based on criteria of WHO (World Health Organization) [15]. However, patients were excluded if: (i) had a history of drug-induced lichenoid lesions [18], (ii) the patient received topical treatments for OLP less than 2 weeks before participating in the study, or systemic treatments 4 weeks before participation [19], (iii) oral tissues affected by OLP revealed loss of pliability or flexibility, (iv) the biopsied sited showed histological signs of epithelial dysplasia.

### Study protocol

#### Clinical steps

For all participants, demographic data (age and gender), and full medical history were recorded followed by clinical examination to prove the presence of OLP in group (A) patients, or its absence in group (B) participants.

## For OLP patients (Group A patients)

### Clinical assessment of OLP clinical severity

Oral lichen planus lesions were clinically assessed using a clinical scoring system [20]. It comprises four distinct categories that encompass all oral criteria of the disease. Each category is assigned a subscore, and the sum of these subscores results in the patient’s final score. These categories are **(i)** Objective Nature of Mucosal Lesions: The rating is determined as follows: 0 for no lesion, 1 for a white keratotic lesion, 2 for atrophy or erosion alone or combined with white lesions, and 3 for ulceration alone or combined with white lesions. **(ii)** Subjective Pain Score: Participants rated their pain on a scale of 0 to 10, which was then categorized into: 0 for no pain, 1 for mild pain (scores 1–3), 2 for moderate pain (scores 4–7), and 3 for severe pain (scores 8–10). **(iii)** Number of Surfaces Affected in the Oral Cavity, Excluding the Gingiva: A score of 0 is given if only 1 surface is affected or if there’s bilateral involvement of the buccal mucosae. A score of 1 is assigned when more than 1 surface or beyond both buccal mucosae are affected. **(iv)** Gingival Involvement as Desquamative Gingivitis: Assign 0 if there is no gingival involvement, 1 if either a narrow band (1 mm) of gingival involvement or a wide band in fewer than 6 teeth is present, and 2 if there is a wide band (> 1 mm) of gingival involvement in more than 6 teeth. The total score ranges from 0 to 9, with 9 indicating the most severe disease and 0 representing complete resolution [20, 21].

### Biopsy

The biopsy site was meticulously chosen to avoid areas completely devoid of epithelium while ensuring the inclusion of keratotic areas and a portion of normal mucosa. Two punch biopsies were carried out using tissue punch (Biopsy Punch HEMC, Noida, India) sized 10 with a diameter of 3.5 mm. The first biopsy was fixed in 10% neutral buffered formalin for histopathological examination to confirm OLP diagnosis. The second biopsy was preserved in Phosphate Buffer Saline (PBS) solution and stored at -80˚ C for testing the antibody level by ELISA test.

## For healthy controls (Group B participants)

Tissue samples from the control group were obtained from healthy uninflamed tissues excised during periodontal surgeries such as crown lengthening and implant surgeries avoiding any teeth affected by periodontal diseases [22]. The specimens were, then, handled and processed for testing the antibody level using the ELISA test; just as described for the second biopsy of group A.

### Laboratory steps

1. Histopathological examination of OLP biopsies: The first biopsy specimen of OLP lesions was paraffin-embedded to be processed for examination. After deparaffinization and rehydration, the specimens were stained with hematoxylin and eosin, examined with a light microscope at 10 to 40 magnifications. Diagnosis of OLP was confirmed according to the clinical and histopathological criteria for OLP set by Kramer et al. 1978, updated by van der Waal & van der Meij 2003 and recently modified by Warnakulasuriya et al. 2021 [14–16].
2. Biochemical analysis (Detection and quantification of Autoantibodies to Dsg3 by ELISA testing): The frozen biopsy was processed for the ELISA test of Dsg3 autoantibodies [Human Anti-Dsg3 antibody (anti-Desmoglein-3 antibody) ELISA Kit, Wuhan, China by Wuhan Fine Biotech Co., Ltd. (430075)]. Tissue homogenates were centrifuged for 20 min at 1000×g at 2–8 °C to remove insoluble impurities and cell debris. The clear supernatant was used for the determination of Dsg3 autoantibodies by ELISA kit according to manufacturer instructions.

The kit is based on sandwich ELISA technology. 100ul of properly diluted samples were added into test sample wells, incubated at 37 °C for 90 min, then washed 2 times with Wash Buffer. A 100ul Biotin-labeled Antigen working solution was added into the wells (standard, test sample, and blank wells). After incubation at 37 °C for 60 min, the plate was washed 3 times with wash buffer. 100ul of HRP-Streptavidin Conjugate Working Solution was added into each well, and the plate was covered and incubated at 37 °C for 30 min. After washing, 90ul Tetramethylbenzidine Substrate was added and incubated at 37 °C in the dark for 10–20 min. Then, 50 µl stop solution was added into each well to stop the reaction.

The level of Dsg3 antibodies were quantified through optical density (OD) measurement: The blank well was taken as zero, and the absorbance OD was measured of each well one by one under 450 nm wavelength, which was carried out within 10 min after the addition of the stop solution. According to the standards’ concentrations and the corresponding OD values, the linear regression equation of the standard curve was calculated. Then according to the OD value of the samples, the concentration of the corresponding sample was calculated.

### Statistical analysis

Numerical data were explored for normality by checking the distribution of data and using tests of normality (Kolmogorov-Smirnov and Shapiro-Wilk tests). Parametric data were presented as mean and standard deviation (SD) values while descriptive statistics for OLP severity scores included median and range values. For parametric data, Student’s t-test was used to compare between the two groups. Spearman’s correlation coefficient was used to study the correlation between age, OD, the concentration of Dsg-3 Autoantibodies, and OLP severity scores. Gender data were presented as frequencies and percentages. The significance level was set at *P* ≤ 0.05. Statistical analysis was performed with IBM SPSS Statistics for Windows, Version 23.0. Armonk, NY: IBM Corp.

#### Sample size

A power analysis was designed to have adequate power to apply a two-sided statistical test of the null hypothesis that there is no difference between the 2 groups regarding the level of Dsg3 autoantibodies in tissue biopsy. By adopting an alpha level of (0.05), a beta of (0.2) (i.e. power = 80%), and an effect size (d) of (1.63) calculated based on the results of a previous study [23]; the predicted sample size (n) was a total of (14) cases (i.e. 7 cases per group). Sample size calculation was performed using G*Power version 3.1.9.7. An extra 6 participants (3 in each group) were selected to compensate for any dropouts during the study.

## Results

The current case-control study included twenty participants-ten per group- to detect the levels of Dsg3 autoantibodies in their tissue biopsies and to compare between them. 

Baseline characteristics. Descriptive statistics of base line characteristics in the two groups are presented in (Table 1).

 For the demographic data, group A (OLP) included two males (20%) and eight females (80%) while group B (control) included three males (30%) and seven females (70%). The participants had a mean age of 50.5 (± 7.8) years for group A and 40.3 (± 4) years for group B. The baseline demographic data showed no statistically significant difference in gender and age distribution among the two groups (*p* = 0.494 and 0.307 respectively). Regarding the baseline clinical data for group A, the patients had mean clinical severity scores of 6 (± 1.1).

**Table 1:** Descriptive statistics of base line characteristics in the two groups

Baseline characteristics	OLP (*n* = 10)	Control (*n* = 10)
Gender [n, (%)]		
Male	2 (20%)	3 (30%)
Female	8 (80%)	7 (70%)
Age in years [Mean, (SD)]	50.5 (7.8)	40.3 (4)

Frequencies (n), percentages (%), mean and standard deviation (SD) values for baseline characteristics in the two groups

### Outcomes

1. Desmoglein-3 autoantibodies levels in tissues. (Table 2)

**Fig. 1:**
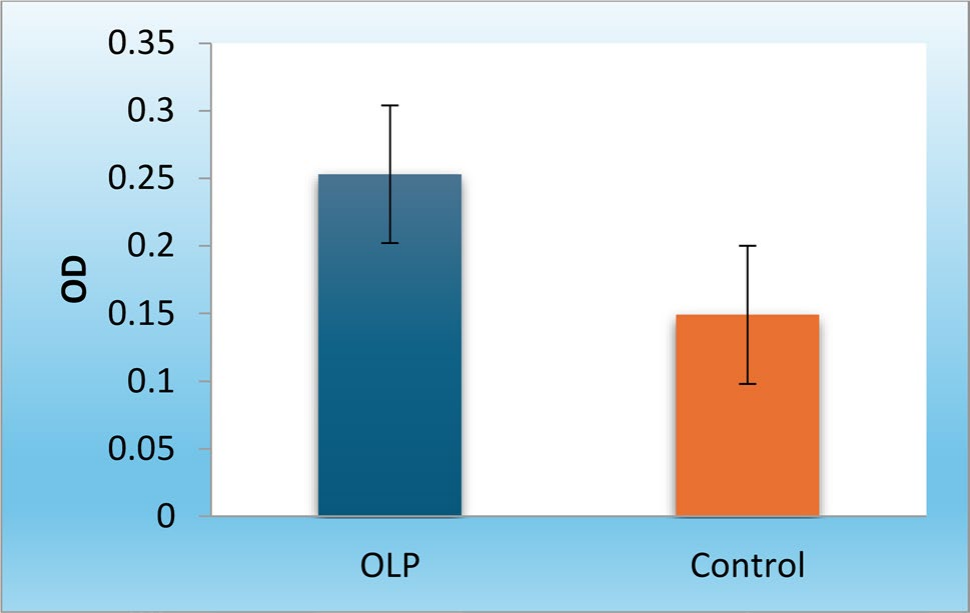
Bar chart representing mean and standard deviation values for OD of Desmoglein-3 Autoantibodies in the two groups

The optical density (OD) of Desmoglein-3 autoantibodies of group A (OLP group) showed a statistically significantly higher mean (P-value < 0.001) compared to group B (control group) shown in Figure 1.

**Fig. 2:**
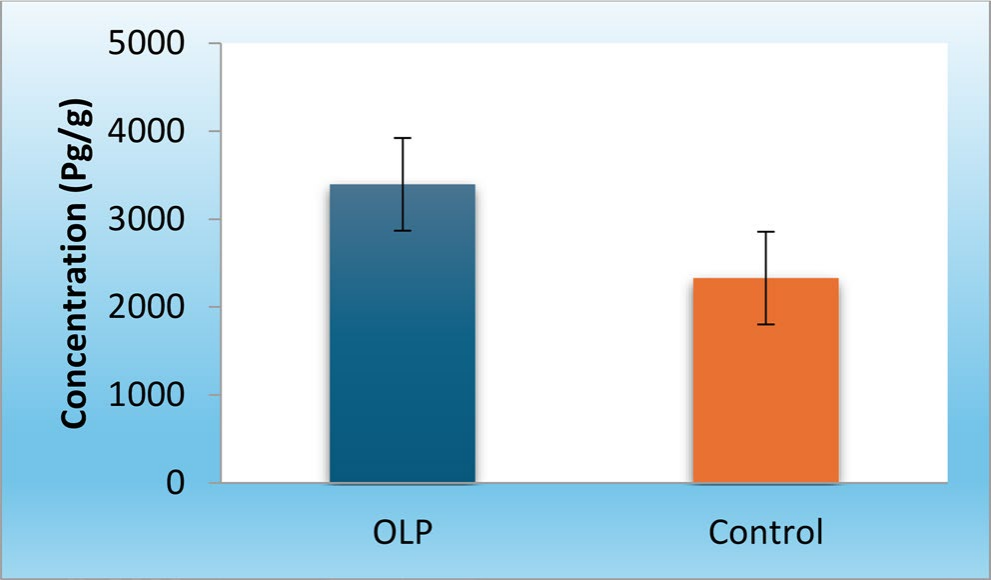
Bar chart representing mean and standard deviation values for the concentration of Desmoglein-3 autoantibodies in the two groups

Accordingly, the concentration of Desmoglein-3 autoantibodies in tissues was calculated; revealing a statistically significantly higher mean in the OLP group compared to the control group (P-value < 0.001) shown in Figure 2.

**Table 2:** Desmoglein-3 autoantibodies levels in the two groups

	Group A [OLP] (*n* = 10)	Group B [Control] (*n* = 10)	*P*-value**
**Optical density** [Mean (SD)]	0.253 (0.051)	0.149 (0.029)	< 0.001*
**Concentration of Dsg-3 autoantibodies (Pg/g)** [Mean (SD)]	3395.4 (526.9)	2329.7 (307.6)	< 0.001*

**Fig. 3:**
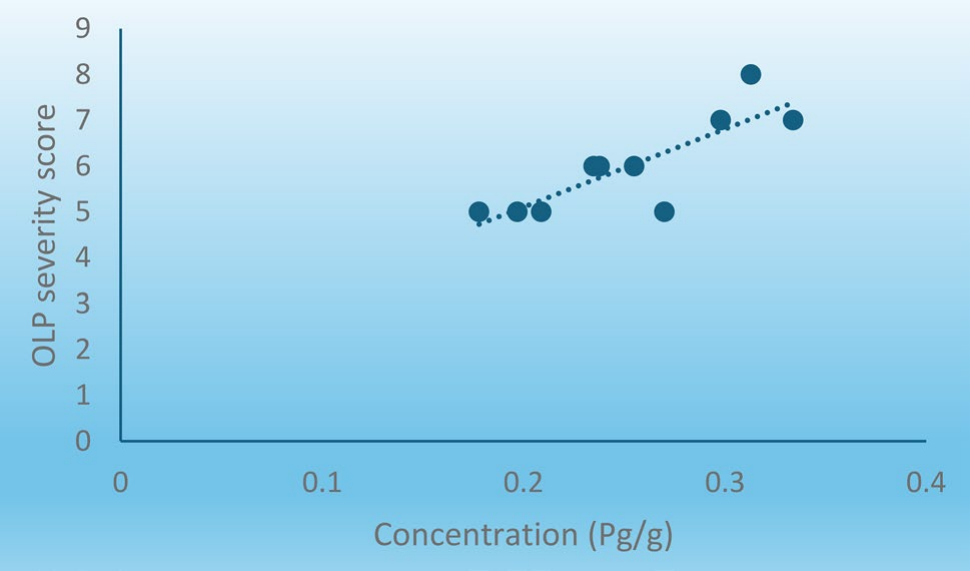
Scatter diagram representing direct correlation between concentration of Desmoglein 3 Autoantibodies and OLP severity score

* Significant at *P* ≤ 0.05; **Calculated using Student’s t-test

2. Correlation between concentration of Desmoglein-3 Autoantibodies and OLP clinical severity. The optical density and concentration of Dsg-3 autoantibodies showed a statistically significant direct (positive) correlation with OLP clinical severity scores (Correlation coefficient = 0.801, P-value = 0.005). In other words, the concentration of Dsg3 autoantibodies was found to increase in cases with higher clinical severity of OLP and vice versa shown in Fig. 3.

## Discussion

The erosive type of oral lichen planus represents a chronic inflammatory disease that manifests as multiple painful erosions and ulcerations of the oral mucosa. The patients suffer from chronic burning sensation with episodes of remission and exacerbation; impairing their quality of life [24, 25]. However, owing to the not yet fully discovered exact pathogenesis of OLP, management aims to induce remission rather than to heal the lesions, resulting in unsatisfactory results [5].

In the quest to discover OLP pathogenesis, Dsg3 autoantibodies were detected in the serum of some erosive OLP cases. However, the previous studies on this topic are limited to retrospective studies and case reports. Furthermore, to date, the literature lacks evidence about the levels of Dsg3 autoantibody in tissues of OLP patients [12]. Thus, the current study was carried out to detect the level of Dsg3 autoantibodies in tissues affected by atrophic/bullous erosive OLP; trying to elucidate if Dsg3 autoantibodies have an actual role in the etiopathogenesis of OLP and correlate it with the disease severity.

To evaluate the clinical severity of OLP in the current study, Elsabagh clinical severity score was used [20]. While numerous OLP scoring systems have been utilized over the past three decades, only few of them are considered to be appropriately valid and none has been examined for accuracy, sensitivity, or specificity. On the other hand, the clinical severity score has been verified to be a valid, reproducible, accurate, and sensitive method for assessing the severity of OLP lesions [26]. Furthermore, it has the advantage of providing a comprehensive assessment, which includes both subjective and objective methods of assessment within it. In addition, it is easy to teach, relatively faster to master, and does not need complicated calculations [20].

After obtaining the demographic data of participants and assessing the clinical severity of the lesions, tissue biopsies were harvested. The present study is unique in detecting Dsg3 autoantibodies in tissue biopsies rather than in serum samples used by all previous studies [12]. Tissue biopsy was favored over serum samples, where OLP-induced tissue damage releases tissue proteins in the blood. The now abnormally circulating damaged tissue proteins (including desmogleins) can stimulate autoantibody release. So, if Dsg3 autoantibodies were detected in the OLP patient`s serum, it cannot be certainly discovered if the autoantibodies were released as a reaction to the already circulating desmogleins, or if the autoantibodies played a role in the disease process. In the first scenario, the antibodies would be available in blood but not in the diseased tissues; while in the second one, the antibodies will circulate in the blood till reaching the target tissues where they can be detected in both blood and tissue samples. Therefore, tissue biopsy is considered a more reliable method to determine the role of the autoantibodies in OLP pathogenesis.

The results of the current study showed that the levels of Dsg3 autoantibodies in patients with atrophic/erosive OLP were significantly higher when compared with healthy controls. Moreover, the concentration of Dsg3 autoantibodies revealed a statistically significant positive correlation with OLP clinical severity scores.

The results of the current study were only contradicted by two previous studies. A case report presented a case where serum Dsg1 and Dsg3 autoantibody levels were 49 and 36 respectively, on first examination. 4 months after the treatment with topical tacrolimus, the levels were slightly reduced to 35 and 27. At 2 years after the treatment, The levels were 46 and 32, respectively. During follow-up visits, no lesions were clinically detected, yet antibody levels remained consistently high [27].

This contrast might be a result of using serum samples in Kinjyo et al., 2015 study, where the source of the autoantibodies cannot be ascertained to be from the oral lesions. The current study overcame this uncertainty by obtaining fresh tissues of OLP lesions based on which, the results were extracted. Therefore, our results can ensure that the source of the autoantibodies is the lesion itself [27].

Furthermore, our results were contrasted by a previous study reporting the absence of an association between the severity of the disease and Dsg3 autoantibodies serum levels in patients with erosive OLP [2]. This contradiction might be due to the different scoring systems used to evaluate the disease severity. In Vahide et al. study, the severity of OLP was assessed by Reticulation, Erosion, and Ulceration scoring system (REU); while in our study, we used a more reliable scoring system, Elsabagh scoring system [2, 20].

On the other hand, the results of the present study regarding Dsg3 autoantibody level came in accordance with the results of a previous retrospective cohort study held on 57 patients with OLP. The level of serum Dsg3 autoantibodies was found to be significantly higher in patients with erosive OLP (82%, *n* = 18/22) compared to healthy control (5%, *n* = 1/20) and those with reticular OLP (20% *n* = 3/15). The results suggested that humoral autoimmunity seems to be involved in the pathogenesis of OLP; while the differences in the serum concentration of Dsg3 autoantibodies suggest that pathological mechanisms in erosive and reticular forms of OLP might not be the same [23].

The current study results also matched the results of a previous case-control study on 35 patients with OLP and 35 healthy controls, who were tested for serum autoantibodies against Dsg3. A significant increase in serum autoantibody to Dsg3 was found in patients with OLP (*P* = 0.00) [28].

In a retrospective cohort study on 22 patients with OLP, positive results for serum Dsg3 autoantibodies were discovered in one case of a severe erosive OLP patient (20 IU/ml; normal range < 7 IU/ml). Thus, they suggested that the more inflammatory the disease, the higher the probability of detecting circulating autoantibodies against epithelial antigens. This finding goes in agreement with the results of the current study; where more severe disease forms were correlated with higher levels of the antibodies [29]. Furthermore, two Japanese cases of erosive OLP were reported with high serum levels of Dsg1 and Dsg3 autoantibodies (34 and 19, normal\7); but it was described that these autoantibodies may be considered as non-pathogenic [30].

Additionally, our findings align with those from a previous retrospective study that investigated the presence of Dsg1 and Dsg3 autoantibodies in patients with various types of OLP, comparing them to cutaneous LP (CLP) patients and healthy controls. The study found that serum levels of Dsg3 autoantibodies were significantly higher in patients with erosive OLP compared to healthy controls. However, this increase was not seen in patients with reticular OLP or CLP. Although the difference was statistically significant, it remained below the cut-off values, rendering it not clinically significant [2].

In agreement with our results, a cross − sectional epidemiological study reported that serum Dsg1 and Dsg3 autoantibodies were high (19% and 16% respectively) in a cohort of 100 OLP patients [13]. Furthermore, a study was performed on twenty adult patients diagnosed with erosive form of OLP; showing similar results as there was an increased level of serum Dsg3 autoantibodies [21.59 (± 11.81)] at baseline. However, with the use of topical Tacrolimus 0.1%, results revealed a statistically significant decrease in the serum level of Dsg3 autoantibodies [1.15 (± 1.03)]. This, also, supports our result that there is a direct correlation between concentration of Dsg3 autoantibodies and OLP severity [31].

Lately, in 2022, in accordance with our results, a case-control study involved 40 cases with OLP and 40 matched healthy controls. The serum levels of Dsg3 autoantibodies in OLP patients [1361.37 (± 300.5)] were found to be significantly higher (*p* < 0.001) than that in normal controls [66.98 (± 66.98)] [32].

However, the results of the current study are limited by the study design as a case-control study can only prove association but cannot prove causation. In other words, the study can prove that Dsg3 autoantibodies are detected in the tissues of OLP and that their activities increase with increasing the severity of the lesions. However, the direction of the effect cannot be concluded; where it is not possible to assure if Dsg3 autoantibodies attacked the tissues first eliciting the inflammatory reaction; or that the inflammatory reaction started first exposing Dsg3 and allowing the autoantibodies to attack them.

Based on all the afore-discussed evidence, the current study can be considered the first to detect the levels of Dsg3 autoantibodies in the tissues of OLP patients; opposed by all previous studies detecting it in serum. This point provides evidence that the detected autoantibodies are certainly originating from the lesions, not elsewhere in the body. Furthermore, the results proved significantly higher levels of autoantibodies in the tissues affected by atrophic/bullous erosive OLP in comparison to healthy controls. This indicates that Dsg3 autoantibodies may be involved in the pathogenesis of OLP. Moreover, it was reported that the levels of the autoantibodies correlated positively with the severity of the lesion. This was interpreted that those autoantibodies had higher levels and higher activity in more severe lesions.

## Conclusions

In conclusion, our study demonstrates that tissues with the atrophic/erosive subtype of OLP have greater levels of Dsg3 autoantibodies than healthy controls. Furthermore, a statistically significant positive correlation exists between the concentration of Dsg3 autoantibodies and the severity scores of OLP. Hence, further investigation is needed to discover the exact role of Dsg3 autoantibodies in the pathogenesis of OLP https://pubmed.ncbi.nlm.nih.gov/26351474/.

## Data Availability

The datasets used in the current study are available from the corresponding author upon request.

## Abbreviations

OLP: Oral lichen planus
DSG: Desmoglein
PBS: Phosphate Buffer Saline
CLP: Cutaneous lichen planus
REU: Reticulation, Erosion and Ulceration scoring system
OD: Optical density
A/BE: Atrophic/bullous-erosive
